# CHKB-AS1 enhances proliferation and resistance to NVP-BEZ235 of renal cancer cells via regulating the phosphorylation of MAP4 and PI3K/AKT/mTOR signaling

**DOI:** 10.1186/s40001-023-01558-w

**Published:** 2023-12-14

**Authors:** Xinglin Chen, Tongtong Zhang, Xiaohan Ren, Yuang Wei, Xu Zhang, Xinyue Zang, Xiran Ju, Chao Qin, Dongliang Xu

**Affiliations:** 1 Urology Centre, Shuguang Hospital Affiliated to Shanghai University of Traditional Chinese Medicine, 528 Zhangheng Road, Pudong New District, Shanghai, 201203 China; 2 Department of Urology, The First Affiliated Hospital of Nanjing Medical University, No. 300, Guangzhou Street, Nanjing, 210029 Jiangsu Province China

**Keywords:** ccRCC, NVP-BEZ235, Drug resistance, Oncology, Urology

## Abstract

Targeted therapy is pivotal in renal carcinoma treatment, and the dual-inhibitor NVP-BEZ235 has emerged as a promising candidate in preliminary studies. Its effectiveness against renal carcinoma and the mechanisms underlying potential resistance, however, warrant further exploration. This study aims to elucidate these aspects, enhancing our understanding of NVP-BEZ235's future clinical utility. To investigate resistance mechanisms, renal cancer cell lines were exposed to progressively increasing concentrations of NVP-BEZ235, leading to the development of stable resistance. These resistant cells underwent extensive RNA-sequencing analysis. We implemented gene interference techniques using plasmid vectors and lentivirus and conducted regular IC50 assessments. To pinpoint the role of LncRNAs, we utilized FISH and immunofluorescence staining assays, supplemented by RNA pull-down and RIP assays to delineate interactions between LncRNA and its RNA-binding protein (RBP). Further, Western blotting and qRT-PCR were employed to examine alterations in signaling pathways, with an animal model providing additional validation. Our results show a marked increase in the IC50 of NVP-BEZ235 in resistant cell lines compared to their parental counterparts. A significant revelation was the role of LncRNA-CHKB-AS1 in mediating drug resistance. We observed dysregulated expression of CHKB-AS1 in both clinical samples of clear cell renal cell carcinoma (ccRCC) and cell lines. In vivo experiments further substantiated our findings, showing that CHKB-AS1 overexpression significantly enhanced tumor growth and resistance to NVP-BEZ235 in a subcutaneous tumorigenesis model, as evidenced by increased tumor volume and weight, whereas CHKB-AS1 knockdown led to a marked reduction in these parameters. Critically, CHKB-AS1 was identified to interact with MAP4, a key regulator in the phosphorylation of the PI3k/Akt/mTOR pathway. This interaction contributes to a diminished antitumor effect of NVP-BEZ235, highlighting the intricate mechanism through which CHKB-AS1 modulates drug resistance pathways, potentially impacting therapeutic strategies against renal carcinoma.

**Supplementary Information:**

The online version contains supplementary material available at 10.1186/s40001-023-01558-w.

## Introduction

Kidney cancer remains a significant health concern worldwide. According to the most recent statistics from the American Cancer Society, in 2023, approximately 81,800 adults in the United States are estimated to be diagnosed with kidney cancer, including 52,360 men and 29,440 women. Additionally, about 14,890 people (9920 men and 4970 women) are expected to die from this disease in the same year [33]. From the perspective of pathology, almost all kidney cancers are derived from the renal tubular epithelium, therefore called renal cell carcinoma (RCC). RCC can be generally divided into three major histological subtypes: clear cell RCC (ccRCC), papillary RCC (pRCC) and chromophobe RCC (chRCC), among which ccRCC is most frequent to see with a proportion of over 80% and an incidence of 2% in adults [13]. Genetically, more than 90% of ccRCC possesses a regular loss or mutation of the von Hippel–Lindau (VHL) gene located on human chromosome 3p, which is considered as the initiating step of ccRCC. With the discovery of genetic mutations in ccRCC, more influential molecules and signaling pathways (e.g., HIF-1/2, PBRM1, SETD1, BAP1 and PI3k/Akt/mTOR) have been revealed to participate in the proliferation, metastasis, and therapeutic targets this disease [16]. In recent years, the role of long non-coding RNAs (lncRNAs) in cancer biology has gained significant attention. These non-coding RNA molecules, previously thought to be non-functional, have now been recognized as key regulators in various biological processes, including cancer development and progression. Studies have demonstrated that lncRNAs can influence gene expression, chromatin remodeling, and signal transduction, thereby playing critical roles in oncogenesis, metastasis, and drug resistance [2, 26].

The growing understanding of genomic features is hopefully to bring specific therapeutic strategies for ccRCC patients. Based on the inspiring findings, the innovation of small molecular inhibitors radically reformed the treatment situations of ccRCC [27]. Today, combined with immunotherapy, there are several alternative small molecular inhibitors for first-line and second-line standard therapies of ccRCC. These drugs could target key factors or genes involved in ccRCC, including VEGF, VEGFR/PDGFR, mTOR and PD-1/PD-L1 [40]. Among these molecular targets, the phosphatidylinositol 3-kinase (PI3K)/Akt/mammalian target of rapamycin inhibitor (mTOR) pathway has proved to be an indispensable driving signaling and associated with tumor progression, treatment response, and clinical outcomes in many types of tumors. Therefore, it confers specific and targetable anticancer therapeutic strategy in human cancers [5, 17].

NVP-BEZ235 (Novartis Pharma) is a synthetic small molecular compound that simultaneously potently inhibits the catalytic activity of class 1 PI3K (by directly competing its ATP-binding site) and mTOR [20]. It has displayed significant anticancer activity against various types of cancer cells including RCC [31, 42], and it is hypothesized that dual PI3K/mTOR inhibitor NVP-BEZ235 is a promising antitumor agent against ccRCC. Nevertheless, no large-scale clinical trials neither solid basic research are available so far to prove its efficacy and safety in real world, let alone its clinical value in treating kidney cancers.

In the present study, by culturing NVP-BEZ235-resistant cell lines, we successfully revealed the potential mechanism of the resistance to NVP-BEZ235 in ccRCC cells and doubted the clinical applicability of this agent. Through the application of RNA-sequencing techniques, cellular experiments, and other molecular biology methods, we discovered a novel signaling pathway mediated by CHKB-AS1, an elevated lncRNA in ccRCC. This pathway significantly affects the activity of the PI3K/Akt/mTOR pathway, thereby regulating the sensitivity of ccRCC cells to NVP-BEZ235. Our work offered new insights into the working disorders of NVP-BEZ235 in kidney cancer and may provide certain support for more large-scale clinical trials in the future.

## Materials and methods

### Open data acquisition

Open data, including the gene expression profile and clinical information of ccRCC patients were obtained from The Cancer Genome Atlas database. Gene Set Variation Analysis (GSVA) was performed using the GSVA package in R software based on the Hallmark gene set.

### Cell culturing and patient samples

Human ccRCC cell lines 786-O and Caki-1 were obtained from the Cell Bank of the Chinese Academy of Sciences (Shanghai, China). The 786-O cells were cultured in RPMI-1640 medium and Caki-1 in McCoy’s 5A medium both with 10% fetal bovine serum (FBS) in an environment of 5% CO_2_ and 37 °C.

A total of 32 paired human ccRCC clinical samples were obtained through radical nephrectomy on patients. Each patient was clinically diagnosed as ccRCC after the surgery by at least two independent pathologists. The informed consents were acquired from patients accordingly. The study was approved by the ethic committee of The First Affiliated Hospital of Nanjing Medical University.

### Construction of NVP-BEZ235-resistant cell lines

The technique used to induce the drug resistance was consistent with our previous study [37]. Parental 786-O and Caki-1 cells were seeded onto the six-well plates. Upon the confluence of 50–60%, the cells were cultured with a replaced medium containing NVP-BEZ235 at a lowest concentration for 48 h and then exposed to a fresh medium without NVP-BEZ235 for 24 h. Cells that stably proliferate were then collected, passaged, and exposed to a 5 μM higher concentration of drug than previous, and the treatment ended when most of the cells no more showed vulnerability to NVP-BEZ235. Then, they are applied as NVP-BEZ235-resistant cell lines for the following research.

### Gene interference

The plasmid vector to overexpress CHKB-AS1 was designed and purchased from Genechem (Shanghai, China) and detailed information is provided in the supplementary materials. The cells were seeded onto 6-well plates and appropriate content of DNA oligo was transfected into cells using Lipofectamine 3000 reagent (Invitrogen, United States). The q-PCR was used to evaluate the overexpressing efficiency. After that, the stably CHKB-AS1-overexpressed cell lines were established and applied for further experiments.

The lentivirus carrying shRNA to silence CHKB-AS1 was designed and purchased from Genechem (Shanghai, China). The cells were seeded onto 6-well plates and appropriate content of RNA oligo was added into the medium. After 24 h, the q-PCR was conducted to evaluate the knocking down efficiency. Finally, the stable knockdown of cells was fulfilled, and the cells were used as shCHKB-AS1 cell lines.

### Quantitative real-time PCR (qRT-PCR) assay

The q-PCR was performed consistent to our previous study [37]. Total RNA was isolated from ccRCC cells or samples by Trizol Reagent (Thermo Fisher Scientific, United States) and reversely transcribed into cDNAs using the reverse transcription kit (Thermo Fisher Scientific, United States) following the manufacturer’s protocol. Then, the qRT-PCR was conducted on a LightCycler 480 II (Roche Diagnostics, Basel, Switzerland) instrument using the SYBR-Green master kit (Vazyme, Nanjing, China). The primers used to amplify CHKB-AS1 were purchased from TsingKe Biotechnology (Shanghai, China), which were designed as 5ʹ-AGGCCAATCAGTTGGCGGAG-3ʹ (forward) and 5ʹ-GCTGGACGGCTCTTCCTTGT-3ʹ(reverse). The primers for β-actin were 5ʹ-CACCATTGGCAATGAGCGGTTC-3ʹ (forward) and 5ʹ-AGGTCTTTGCGGATGTCCACGT-3ʹ (reverse). Each qRT-PCR was performed in triplicate, and β-actin was utilized as a control to normalize gene expression.

### Western blotting

The Western blotting assay was performed consistent to our previous study [37]. Total proteins were extracted from ccRCC cells using radioimmunoprecipitation assay (RIPA) buffer. Proteins were separated by 10% sodium dodecyl sulfate–polyacrylamide gel electrophoresis (SDS-PAGE) and transferred to a polyvinylidene difluoride (PVDF) membrane. After blocked within skim milk for 2 h, the membranes were incubated overnight with the primary antibody (Cell Signaling Technology, United States) at 4 °C. Then, the secondary antibody (Cell Signaling Technology, United States) was used to incubate the membranes for 2 h. GAPDH expression was used as a loading control. Finally, the protein bands were visualized with an enhanced chemiluminescence (ECL) detection system (Thermo Fisher Scientific, Rochester, NY, United States).

### Cell viability detection

The cell viability assays were performed consistent to our previous study [37]. To detect the viability of cells, Cell Counting Kit-8 (CCK-8) assay and colony formation assay were performed. First, 2 × 10^3^ cells were seeded onto 96-well plates and treated with certain medium. Different concentrations of drug were added into different wells, and the cells were treated for 24–48 h. At the time of 24, 48, 72 and 96 h, ten microliters of CCK8 reagent (Solarbio, Japan) were added into the wells, and the cells were incubated in an environment of 37◦C and 5% CO_2_ for 1 h. At last, the OD450 of cells was detected using an Epoch Microplate Spectrophotometer (BioTek Instruments, Inc., United States), and the cell viability and 50% inhibiting concentration (IC50) could be calculated accordingly.

Then, 1 × 10^3^ cells were seeded onto the 6-well plates. NVP-BEZ235 was added into the wells, and the cells were cultured for 6–10 days. The medium was changed every 3 days. After that, the cells were fixed with 4% formaldehyde for 15 min and stained with 0.1% crystal violet for 20 min before counting the number of colonies.

### Fluorescence in situ hybridization (FISH) and immunofluorescence staining

Fluorescent probe specific to CHKB-AS1 was applied to determine the subcellular location of CHKB-AS1 in ccRCC cells using a FISH Kit (RiboBio, China). First, the cells were fixed with 4% paraformaldehyde for 10 min and permeabilized for 5 min with PBS containing 0.5% Triton X-100. Then, cells were treated with a FISH probe, incubated overnight at 37 °C. Finally, they were washed with a 4 × saline sodium citrate (SSC) buffer for 5 min and a 1 × SSC buffer for 5 min. The cell nucleus was stained with 4,6-diamidino-2-phenylindole (DAPI).

For the immunofluorescence assay, cells were blocked for 1 h using an immunostaining blocking solution (Beyotime, China). They were then incubated with the primary antibody overnight and treated with fluorescent secondary antibody for 1 h in darkness. DAPI was applied for nuclear staining as the control. Finally, the cells were observed under a confocal fluorescence microscope (Leica, German).

### RNA pull-down and mass spectrometry analysis

The RNA pull-down assay was performed to discover potential RNA binding protein (RBP) to Lnc-CHKB-AS1 using the Pierce Magnetic RNA-Protein Pull-Down Kit according to the manufacturer’s protocol (Thermo Fisher Scientific, United States). The biotin-labeled pull-down probe targeting CHKB-AS1 was designed and synthesized by Ribobio Technology (Guangzhou, China). The protein eluted from RNA pull-down assay was then subjected to the mass spectrometry analysis with the help of BioProfile (Shanghai, China).

### RNA binding protein immunoprecipitation (RIP) assay

The RNA immunoprecipitation (RIP) assay was performed using a RIP Kit according to the manufacturer’s protocol (Millipore, USA). To begin with, cells were lysed with RIP lysis buffer containing inhibitors of protease and RNase. Next, they were incubated with anti-MAP4 or anti-FLAG antibodies which had been premixed with the magnetic beads overnight at 4 °C. Digested with the proteinase K buffer, the immunoprecipitated RNA was obtained and detected using qRT-PCR.

### In vivo xenograft model

Nude mice were purchased from the Animal Center of Nanjing Medical University (Nanjing, China) for the generation of xenograft model. For the tumorigenicity, parental or resistant ccRCC cells that were stably transfected with OE-CHKB-AS1 or sh-CHKB-AS1 and control cells were subcutaneously injected into the left and right armpits of the mice, respectively. Meantime, subgroup mice were treated with certain concentration of NVP-BEZ235 or DMSO in a weight-dependent manner via intravenous injection. The tumor volume and weight were regularly measured and recorded every 5 days. At the 30th day since the injection, the mice were killed, and the xenograft tumors were excised for the measurement of volume and weight.

### Statistical analysis

All statistical analyses were conducted using the SPSS software version 19.0. Representative analyses included the Student’s *t*-test (two-tailed), Kaplan–Meier analysis, and the log-rank test. A *P*-value < 0.05 was considered statistically significant for each test. Each assay was performed in three times.

## Results

### Identification of LncRNA-CHKB-AS1 as the hub gene involved in NVP-BEZ235 resistance

**Fig. 1:**
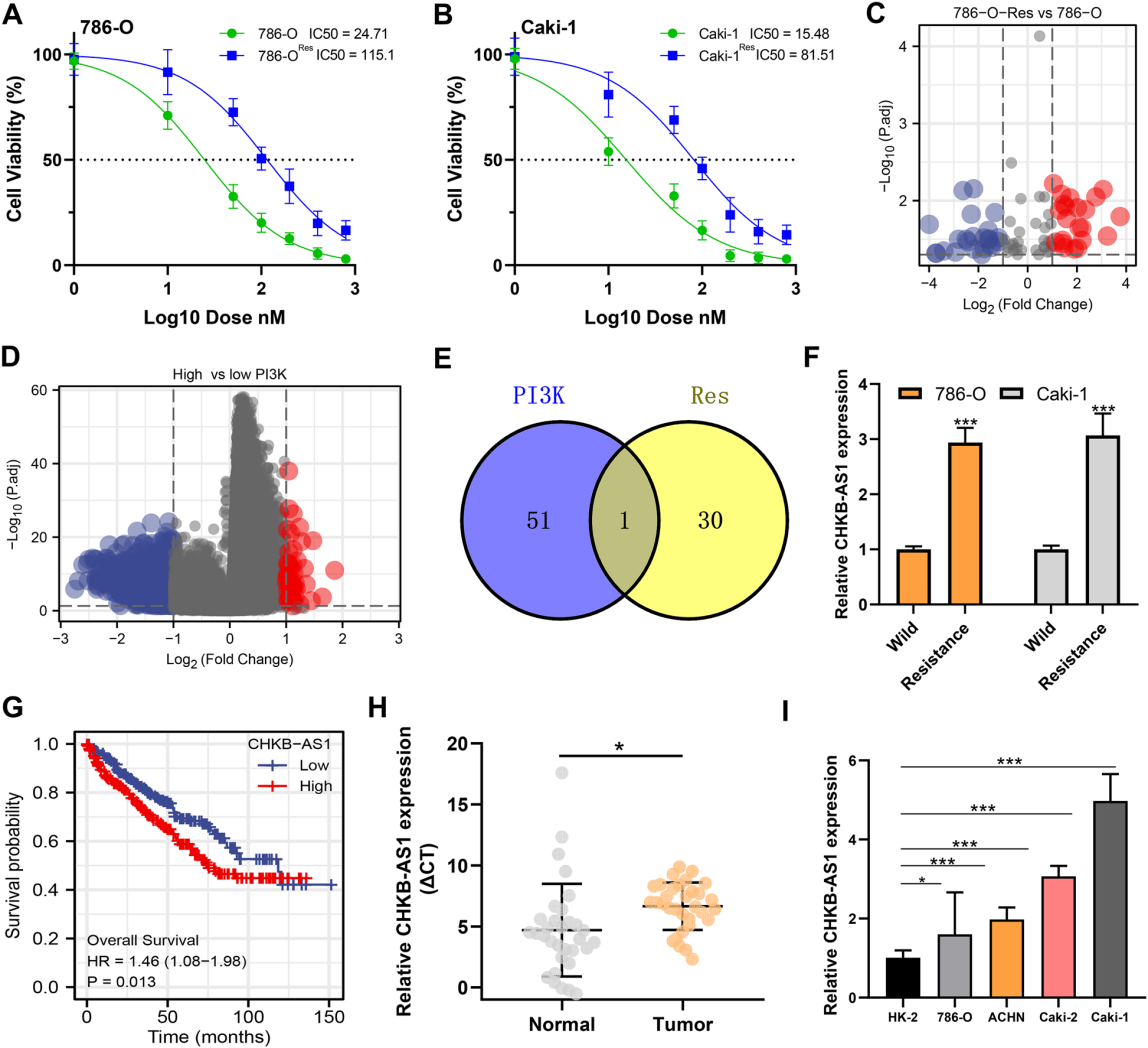
CHKB-AS1 participated in the formation of NVP-BEZ235 resistance. **A**, **B** IC50 suggested the construction of stably resistant 786-O and Caki-1 cell lines. **C**–**E** RNA-sequencing revealed the hub genes involved in the resistance. **F** CHKB-AS1 level in resistant and parental 786-O and Caki-1 cells. **G** Kaplan–Meier’s survival curves of ccRCC patients in low and high CHKB-AS1 groups. **H** CHKB-AS1 level in 32 paired ccRCC samples. **I** CHKB-AS1 level in HK-2 cells and ccRCC cells

ccRCC cell lines 786-O and Caki-1 were cultured under the treatment of NVP-BEZ235 for nearly 6 months, and the results of CCK8 assays finally indicated the acquirement of drug resistance in cells (Fig. 1A, B). According to previous studies, the fold-change of IC50 (half-maximal inhibitory concentration) > 2 was considered to be NVP-BEZ235-resistant cell lines (designated as 786-O-Res and Caki-1-Res) [29, 37]. To discover underlying mechanisms causing drug tolerance, the 786-O-Res cells were subjected to mRNA and LncRNA sequencing analysis. By comparing expressing levels of all mRNAs and LncRNAs, 86 lncRNAs were identified as differentially expressed lncRNAs (DELs) between sensitive and resistant cells (Fig. 1C and Additional file 1). Meantime, DEGs were identified between PI3k-activated and PI3k-inactivated cells according to PI3k levels, as shown in the Volcano plot and the CHKB-AS1 was identified (Fig. 1D). Also, we found that the PI3K/AKT/mTOR signaling was activated in the patients with high CHKB-AS1 expression (Additional file 2: Fig. S1A). The comparison between two groups of DEGs revealed the overlapping gene lncRNA-CHKB-AS1 in the Venn diagram, and it was determined as hub gene in the following research (Fig. 1E). First, validation of the aberrant expression of CHKB-AS1 was performed accordingly. CHKB-AS1 displayed significant overexpression in 786-O-Res cells and Caki-1-Res cells compared to the parental cells (*p* < 0.05) (Fig. 1F). The Kaplan–Meier curves showed that patients with high CHKB-AS1 expression had significantly worse survival outcomes compared to the low CHKB-AS1 group (Fig. 1G). In paired samples of 32 ccRCC patients, CHKB-AS1 showed significantly higher expression in tumors than normal tissues (*P* < 0.001, Fig. 1H). Then, results of qRT-PCR showed the mRNA level of CHKB-AS1 was significantly elevated in RCC cells lines compared to normal tubular epithelial cells HK-2 (*p* < 0.05) (Fig. 1I). These results preliminarily established CHKB-AS1 as a vital gene in NVP-BEZ235 resistance in ccRCC.

### CHKB-AS1 promoted NVP-BEZ235 resistance through activating phosphorylation of PI3k/Akt/mTOR pathway

**Fig. 2:**
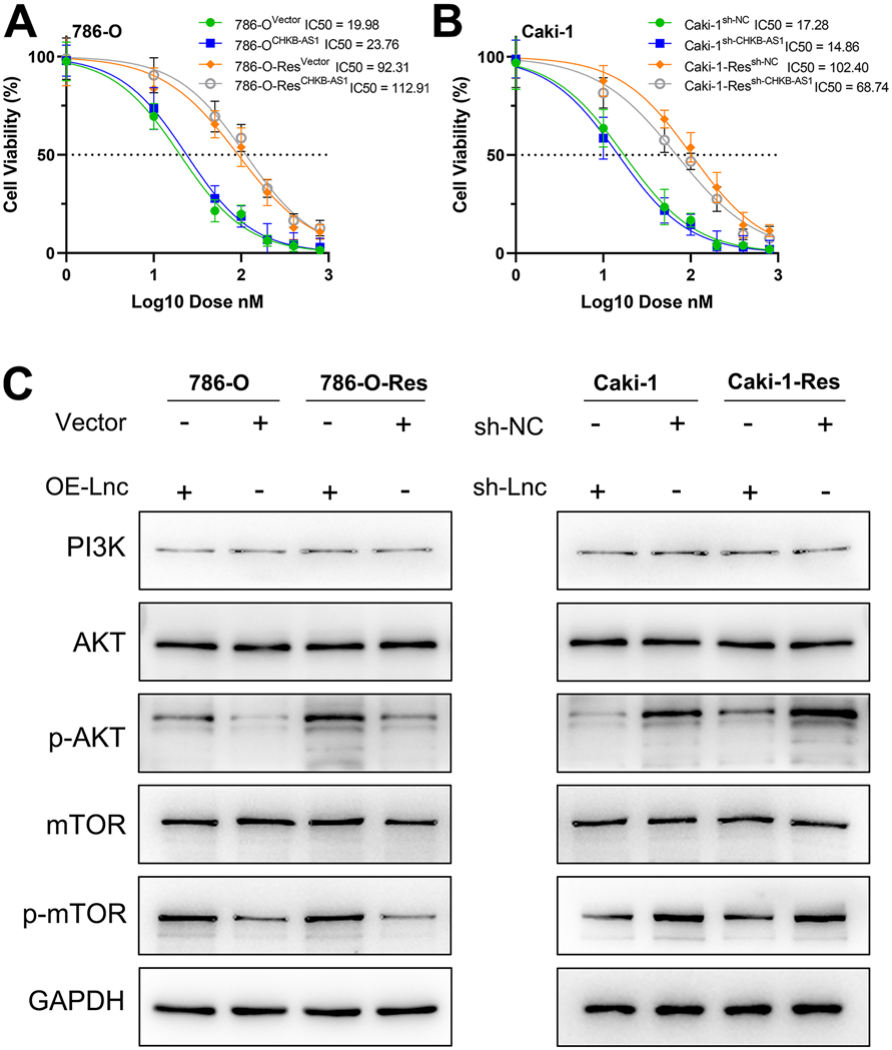
CHKB-AS1 post-transcriptionally modified PI3k/Akt/mTOR pathway. **A** IC50s of parental and resistant 786-O cells transfected with plasmid vectors. **B** IC50s of parental and resistant Caki-1 cells transfected with lentivirus. **C** Alterations of PI3k/Akt/mTOR signaling and its phosphorylation level in parental and resistant ccRCC cells

To verify the facilitating role of CHKB-AS1 in drug resistance inducement, plasmid vectors to overexpress CHKB-AS1 and shRNAs to knockdown CHKB-AS1 were transfected into ccRCC cells, and the proliferative abilities of NVP-BEZ235 treated cells were detected accordingly (Additional file 2: Fig. S1B–I). The results of inhibitory assays showed that CHKB-AS1 overexpression remarkably enhance the resistant capabilities of both 786-O-Res and parental 786-O cells, especially in the former, with IC50 reached to 112.91 nM (Fig. 2A). Similarly, it could be told from the curves that CHKB-AS1 knockdown weakened the tolerance to NVP-BEZ235 of Caki-1 and Caki-1-Res cells, with IC50 decreased to 14.86 nM and 68.74 nM, respectively. For the 786-O cell line overexpressing CHKB-AS1, the IC-50 value increased from 19.98 to 23.76, representing an approximate 18.92% increase. In the case of the 786-O-Res cell line, with CHKB-AS1 overexpression, the IC-50 value rose from 92.31 to 112.91, indicating an approximate 22.32% increase. Conversely, in the Caki-1 cell line, with CHKB-AS1 knockdown, the IC-50 value decreased from 17.28 to 14.86, showing an approximate 14.00% decrease. Lastly, in the Caki-1-Res cell line, also with CHKB-AS1 knockdown, the IC-50 value dropped from 102.40 to 68.74, a significant approximate 32.87% decrease (Fig. 2B). These results preliminarily illustrated that CHKB-AS1 imparts significant resistance to NVP-BEZ235 in RCC cells in vitro, warranting a more comprehensive exploration in subsequent research. Next, we enquired whether CHKB-AS1 functioned through regulation on PI3K/Akt/mTOR signaling pathway. Therefore, the effects of CHKB-AS1 on associated proteins were detected using Western blotting. As shown in Fig. 2C, CHKB-AS1 level mainly affected the protein levels of *p*-Akt and p-mTOR, instead of PI3K, Akt and mTOR in cells. For one thing, in parental 786-O cells, overexpressing CHKB-AS1 could significantly upregulate the protein level of p-Akt and p-mTOR, and the same results were observed in 786-O-Res cells as well. For the other, in parental Caki-1 cells and Caki-1-Res cells, knocking down CHKB-AS1 achieved the equivalent effects on p-Akt and p-mTOR levels. These results proved that CHKB-AS1 probably alleviated the cells response to NVP-BEZ235 by positively regulating the PI3K/Akt/mTOR pathway, especially the activating forms of key proteins namely phosphorylated Akt and mTOR (i.e., p-Akt and p-mTOR).

### CHKB-AS1 promoted tumor progression via countering NVP-BEZ235 efficacy

**Fig. 3:**
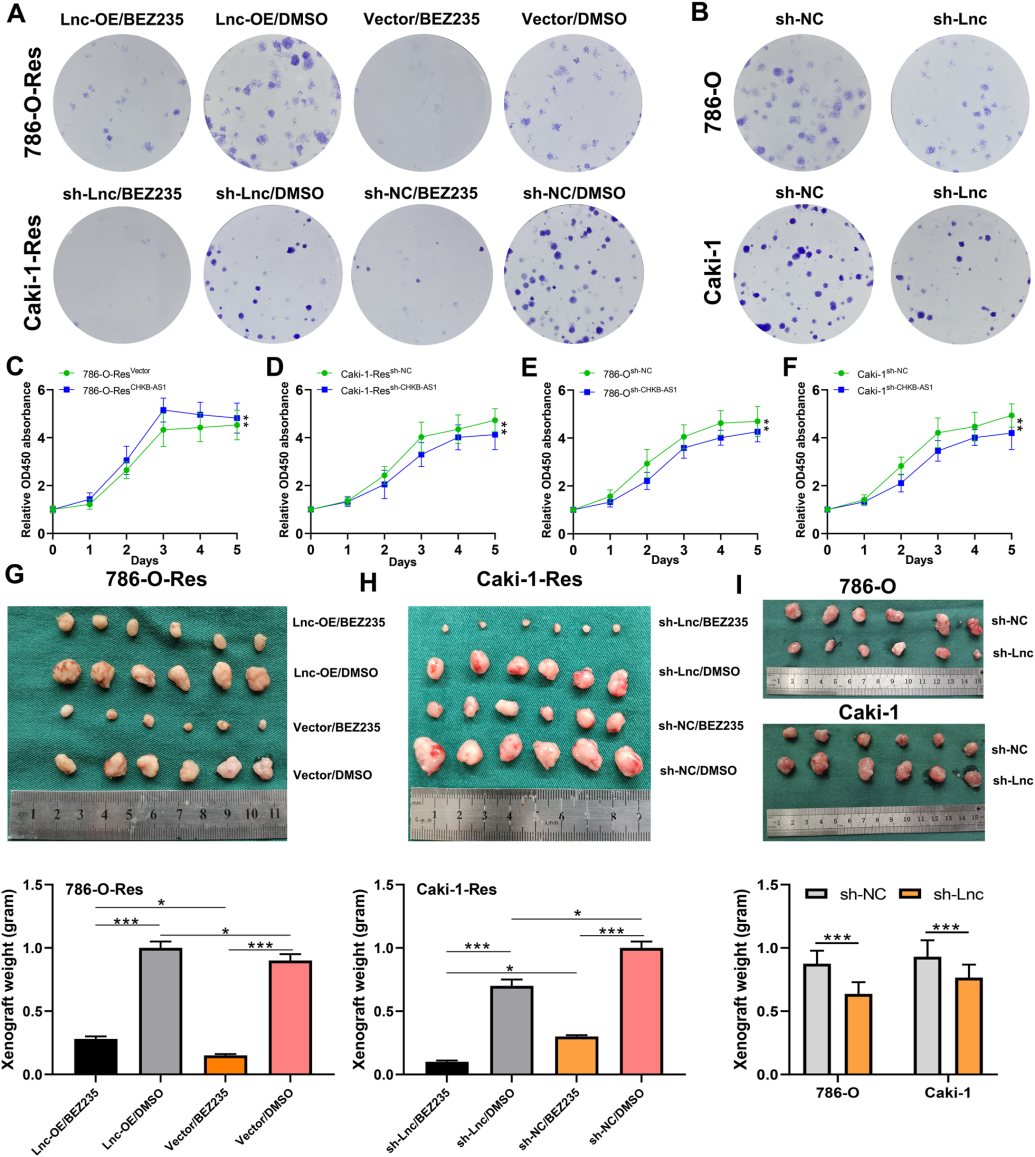
Prominent role of CHKB-AS1 in promoting tumor growth. **A**, **B** Results of colony formation assays on resistant (**A**) and parental (**B**) cells transfected with plasmid vector or lentivirus under NVP-BEZ235 treatment. **C**–**F** Results of CCK8 assays on resistant and parental cells transfected with plasmid vector or lentivirus. **G**–**I** Volumes and weights of tumors extracted from mice injected with cells after genetic interference

Subsequently, in vitro cellular functional assays and in vivo animal experiments were conducted to explore the pro-tumor effects of CHKB-AS1 under the treatment of NVP-BEZ235. Plasmid vector to overexpress CHKB-AS1 and lentivirus to knock down CHKB-AS1 were transfected into 786-O-Res, 786-O, Caki-1 and Caki-1-Res cells, respectively. In overexpression groups, results of colony formation showed that when exposed to NVP-BEZ235 for 24 h, higher CHKB-AS1 level gave 786-O-Res cells significantly stronger survival ability (Fig. 3A). In knockdown groups, equivalent results were observed as well (Fig. 3A, B). Similarly, CCK8 assays showed as the CHKB-AS1 can make 786-O-Res cells gain stronger proliferative ability (Fig. 3C–F). In vivo, subcutaneous tumorigenesis exhibited similar results. First, by comparing the NVP-BEZ235-treated group and DMSO-treated group, the distinct volume of tumors proved that NVP-BEZ235 could enormously weaken the growth of subcutaneous tumors as its function indicated. Then, compared to the control groups, tumors of nude mice in CHKB-AS1-overexpressing group exhibited significantly larger macroscopic volume and actual weight under the treatment of NVP-BEZ235. Consistently, tumors of CHKB-AS1-knockdown group had distinctly smaller volume and weight, when compared to the control group. These results proved that NVP-BEZ235 could exert prominent anti-tumorigenesis efficacy on ccRCC in the relative absence of CHKB-AS1 in vivo (Fig. 3G–I).

### Identification of MAP4 as the key RNA binding protein of CHKB-AS1

**Fig. 4:**
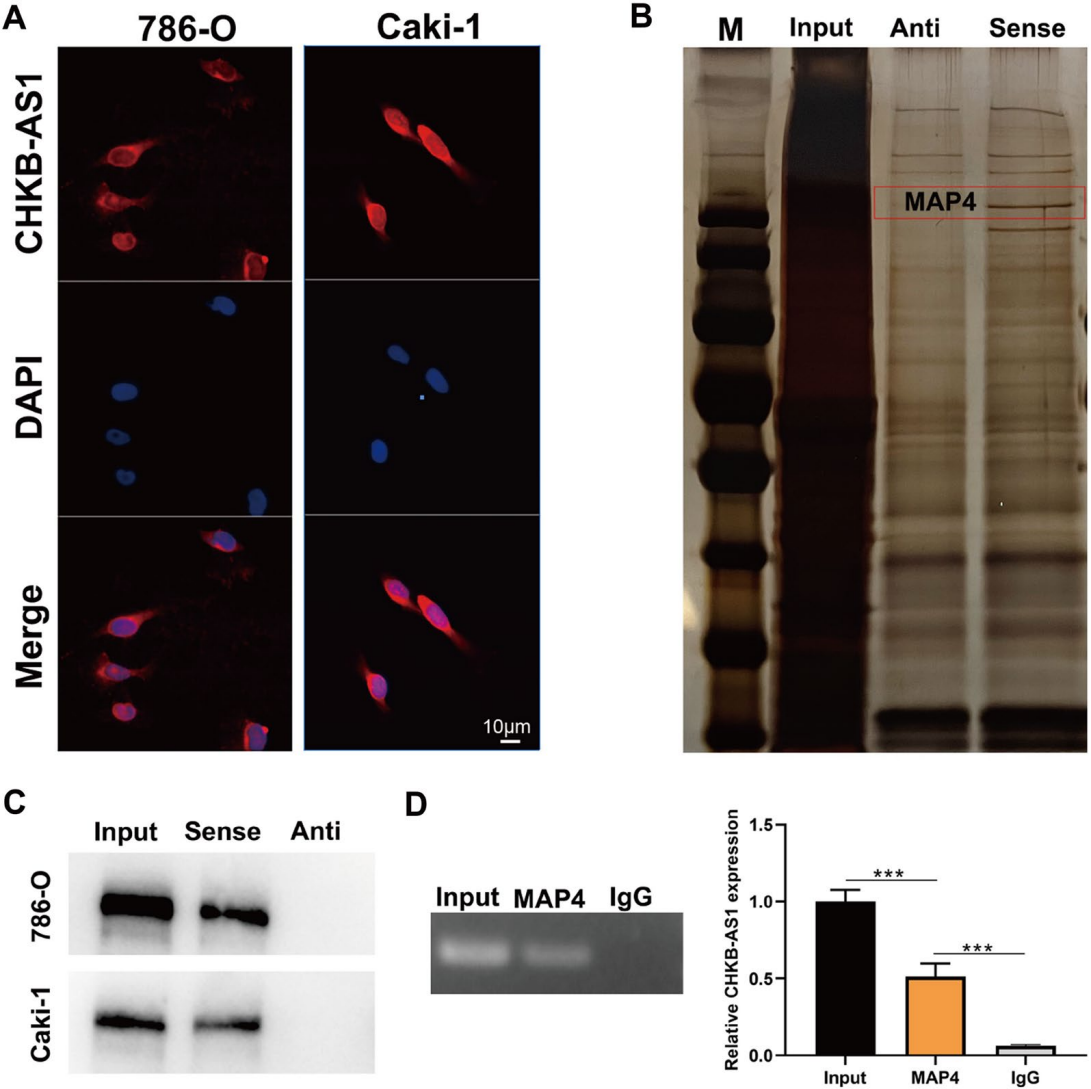
MAP4 was the vital RBP for CHKB-AS1 functioning. **A** Subcellular localization of CHKB-AS1 through immunofluorescence staining. **B**–**D** Results of RNA pull-down assay (**B**), Western blot (**C**), and RIP assay (**D**) to identify MAP4 as RBP to CHKB-AS1

Next, specific molecular regulation of CHKB-AS1 on PI3k/Akt/mTOR pathway was investigated using multiple methods. First, results of immunofluorescence staining showed that CHKB-AS1 was localized and functioning in the cytoplasm instead of nucleus, and it exhibited stronger activity in Caki-1 cells compared to 786-O cells (Fig. 4A). To discover potential RNA-binding protein (RBP) that interacted with CHKB-AS1, RNA pull-down assay was conducted accordingly. The results of RNA pull-down SDS-PAGE revealed a number of differentially expressed proteins between the Anti-group and Sense-group, among which the protein of around 240kD showed distinct gap (Fig. 4B). Then, RNA pull down-mass spectrometry (MS) was used to analyze differentially expressed proteins to screen for potential key protein. After filtration, the protein named microtube associated protein 4 (MAP4) came into notice for its abundance and association with PI3k/Akt/mTOR pathway. WB verified the results of mass spectrometry (MAP4) (Fig. 4C). After that, the binding relationship between CHKB-AS1 and MAP4 was further validated through RNA binding protein immunoprecipitation assay (RIP). The results of qRT-PCR showed again that MAP4 could bind to CHKB-AS1 (*p* < 0.001, Fig. 4D). These results revealed the direct binding relationship between MAP4 and CHKB-AS1, probably through which CHKB-AS1 exerted positive influence on the activation of PI3k/Akt/mTOR pathway.

### CHKB-AS1 restrained MAP4 phosphorylation to alleviate NVP-BEZ235 lethality via binding to N-terminal domain

**Fig. 5:**
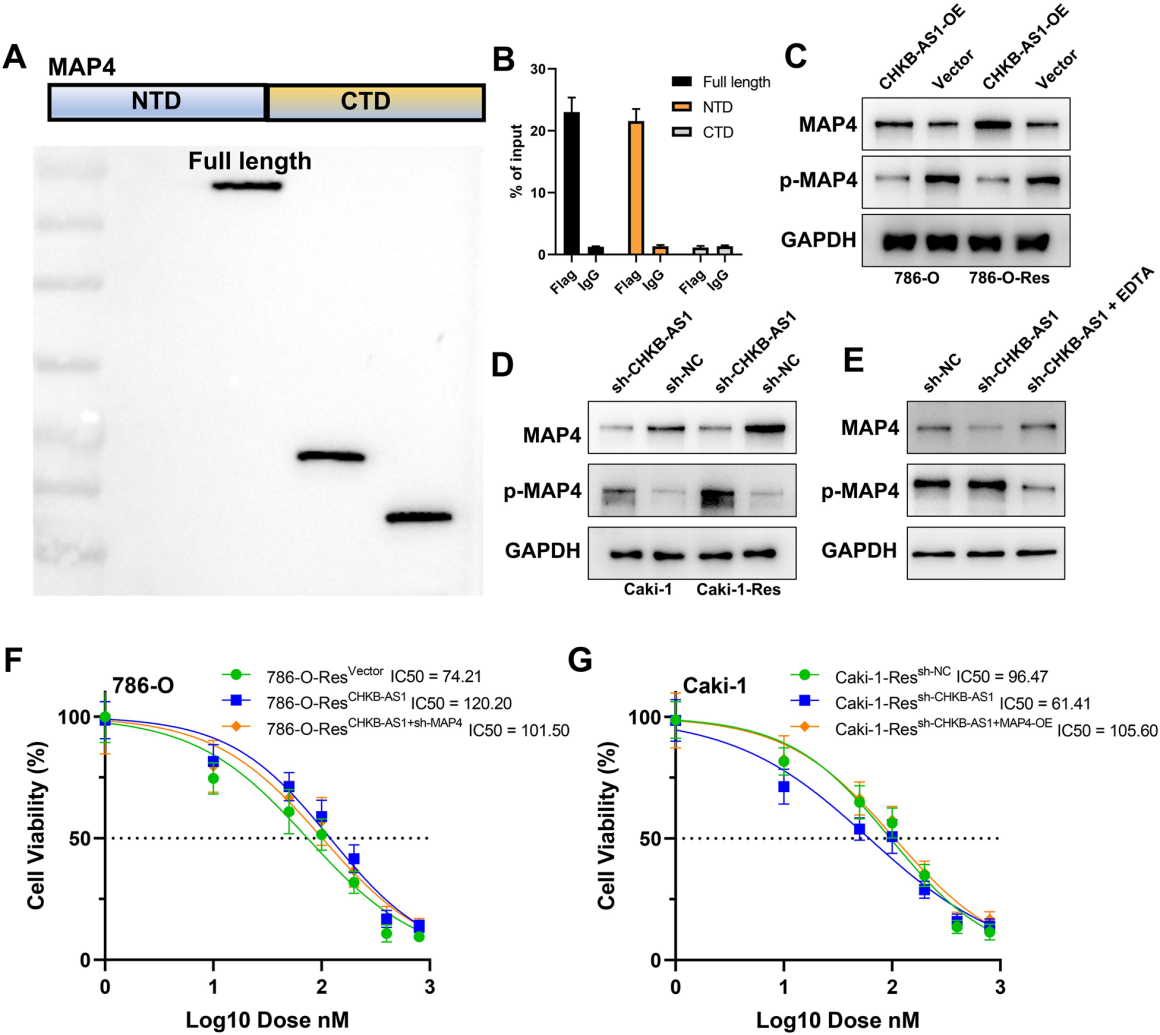
CHKB-AS1 downregulated the phosphorylation of MAP4 to promote drug resistance. **A**, **B** CHKB-AS1 bound to MAP4 in the N-terminal domain. **C**, **D** Western blot showed that CHKB-AS1 prevented MAP4 from being phosphorylated in parental and resistant cells. **E** Phosphorylation of MAP4 might be responsible for the inhibition effect of MAP4 brought by the knockdown of CHKB-AS1. **F**, **G** CCK8 assay showed that CHKB-AS1 up-regulated MAP4 insensitized cells to NVP-BEZ235

To further explore the interactive effects of CHKB-AS1 on MAP4, we constructed plasmid vectors carrying amino acid sequences of N-terminal domain (NTD) and C-terminal domain (CTD) of MAP4 protein with incorporation of Flag protein. After transfecting the vectors into ccRCC cells, the expression and purification of protein domains was detected using Western blotting (Fig. 5A). The result showed the dominant abundance of Flag-tag in NTD group, which could be told that MAP4 bound to CHKB-AS1 through the N-terminal domain (Fig. 5B). In the following research, we transfected shRNA and vectors into ccRCC cells to knockdown and overexpress CHKB-AS1, respectively. In 786-O cell line, the results of Western blot showed that overexpressing CHKB-AS1 increased the MAP4 level while decreased the phosphorylation of MAP4, no matter in parental or NVP-BEZ235 resistant cells. Meantime, CHKB-AS1 knockdown reversely activated the p-MAP4 while downregulated the MAP4 expression in parental and resistant Caki-1 cells (Fig. 5C, D). Ethylene diamine tetraacetic acid (EDTA) can act as a phosphorylation inhibitor [11]. We found that the EDTA can significantly decrease the phosphorylation of MAP4 and reverse the inhibition effect of MAP4 brought by the knockdown of CHKB-AS1 (Fig. 5E). These results indicated that through binding to the NTD of MAP4, CHKB-AS1 potentially suppressed the phosphorylating modification of MAP4 so as to maintain its protein level. Similarly, we observed the NVP-BEZ235 sensitivity of resistant cells when treated with genetic alterations of MAP4. As shown in Fig. 5F, G, the results of CCK8 assay indicated that knocking down MAP4 enhanced the response of 786-O-Res cells to NVP-BEZ235 (IC50 ratio = 101.50:120.20), while MAP4 overexpression granted Caki-1-Res cells more resistance to the agent (IC50 ratio = 105.60:61.41). The results suggested that dephosphorylated MAP4, not p-MAP4, was favorable to the resistance formation of RCC cells.

### CHKB-AS1 promoted NVP-BEZ235 resistance through activating phosphorylation of PI3k/Akt/mTOR pathway via MAP4

**Fig. 6:**
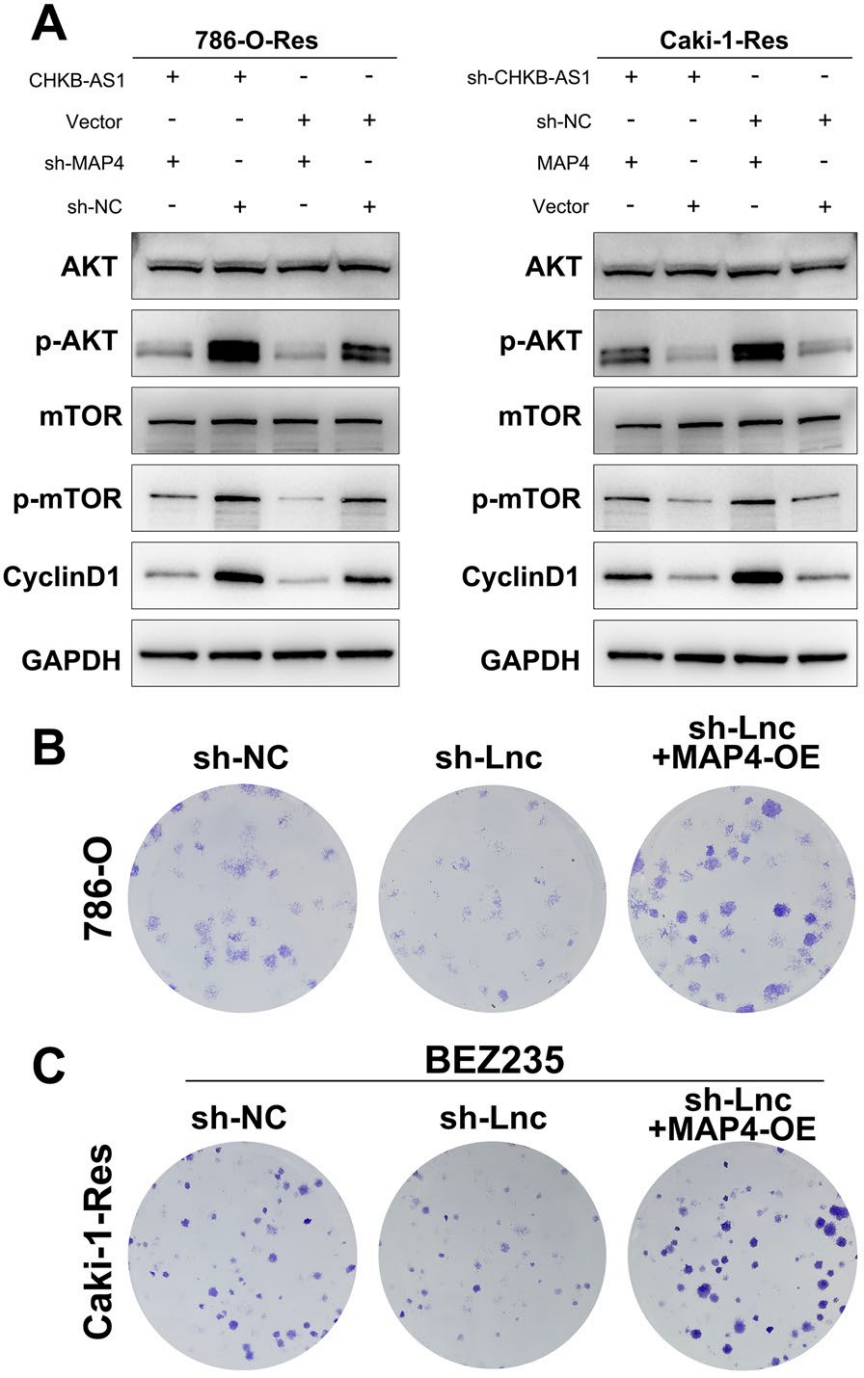
CHKB-AS1 affected drug sensitivity through PI3k/Akt/mTOR signaling in a MAP4-dependent manner. **A** Western blot showed that CHKB-AS1 and MAP4 expression significantly regulated the phosphorylated activation of PI3k/Akt/mTOR signaling. **B**, **C** MAP4 can enhance the proliferation and NVP-BEZ235 resistance through colony formation assay

**Fig. 7:**
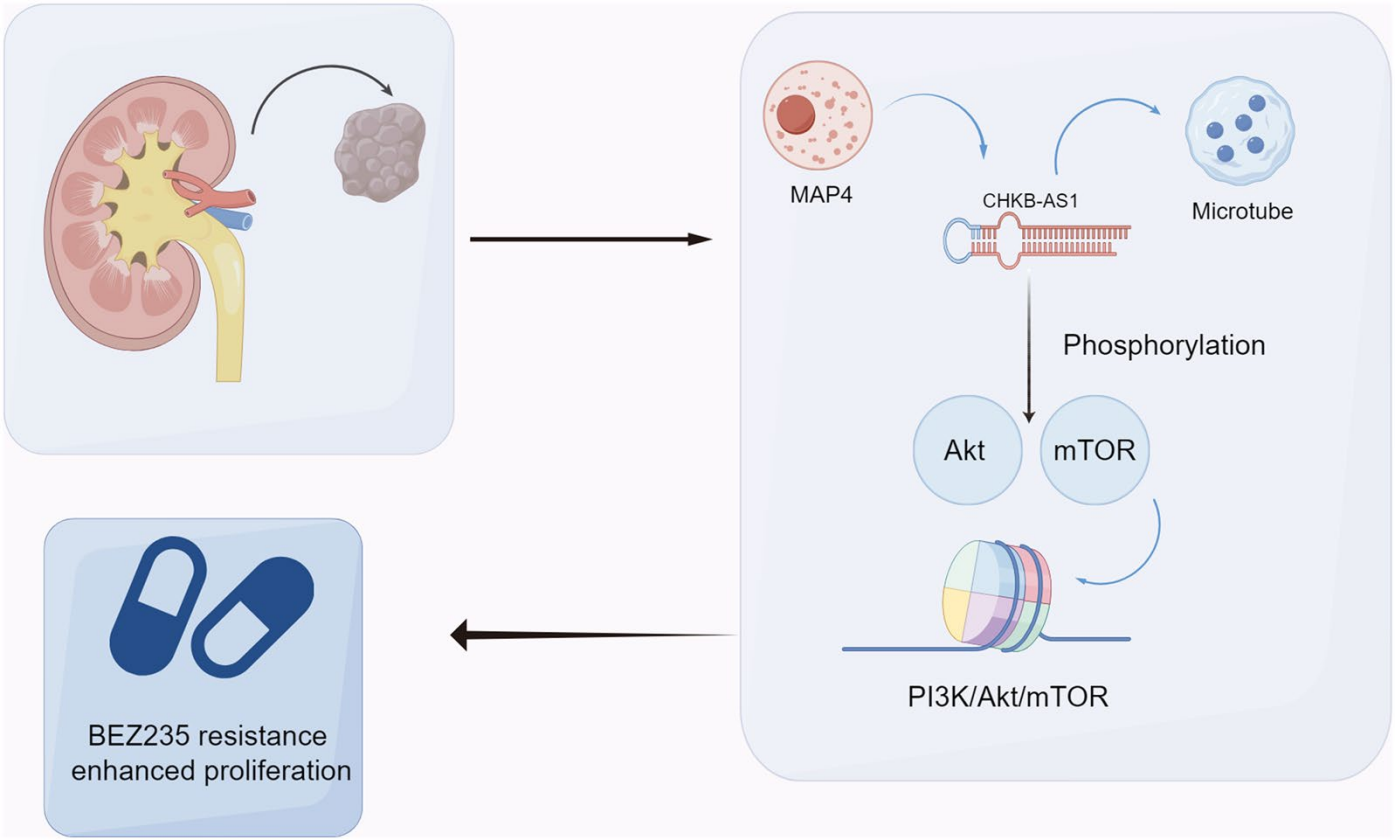
The schematic model of the mechanisms for CHKB-AS1 regulating NVP-BEZ235 sensitivity in ccRCC

Ultimately, we enquired whether the CHKB-AS1-MAP4 axis functioned on PI3k/Akt/mTOR to promote the NVP-BEZ235 resistance formation as conjectured. Plasmid vectors and shRNA were employed to fulfill gene overexpression and silence, and Western blotting was used to detect the alteration of key protein levels. As the results indicated in Fig. 6A, in conditions of no matter overexpressing CHKB-AS1 or not, silencing MAP4 using shRNA led to the significant downregulation of p-Akt and p-mTOR, while the levels of Akt and mTOR were not affected. Similarly, under the circumstance of silencing CHKB-AS1, the downregulation of p-Akt and p-mTOR levels could be significantly reversed by the overexpression of MAP4 gene, which resulted in the apparent phosphorylated modifications of Akt and mTOR. Besides, the protein level of cyclin D1 also displayed a positive trend with MAP4 alteration, indicating the significant activation of upstream pathway PI3k/Akt/mTOR. Taken together, these results proved that by preventing MAP4 from being phosphorylated, CHKB-AS1 stabilized MAP4 protein level which activated the phosphorylation of downstream molecules Akt and mTOR in PI3k/Akt/mTOR pathway. In the 786-O cells, we found that the inhibition of CHKB-AS1 can significantly inhibit cell proliferation ability, while overexpression of MAP4 in sh-CHKB-AS1 cells can enhance the proliferation ability (Fig. 6B). Moreover, in the Caki-1-Res cell treated with NVP-BEZ235, inhibition of CHKB-AS1 can significantly hamper the NVP-BEZ235 resistance, yet overexpression of MAP4 in sh-CHKB-AS1 cells can enhance the NVP-BEZ235 resistance (Fig. 6C). The aberrant activation of PI3k/Akt/mTOR pathway exerted promotive effects on targeted genes involved in cell cycle and growth, cellular metabolism, and protein translation. As a result, the theoretical antitumor effect of dual PI3k/Akt/mTOR inhibitor NVP-BEZ235 was strongly repressed, manifesting as the resistant phenotype in RCC cells. Combining the findings in our study and previous research [42], a schematic diagram was developed to illustrate the underlying mechanism (Fig. 7).

## Discussion

Over years, maximum surgical excision of primary tumors has been the optimal way to treat localized RCC. However, patients of high-stage and advanced RCC still do not have satisfactory long-term survival. As the fundamental understanding of molecular mechanisms of RCC improves, multiple agents are available for RCC patients now. Generally, they developed from the nonspecific immune approach (cytokines and IFN-α) to small molecular targeted drugs (multiple TKIs), and to targeted immunotherapy nowadays (PD-1/PD-L1 inhibitors) [15]. The intrinsic genetic mutation of RCC, which is von Hippel–Lindau (VHL) gene inactivation or deletion, naturally resulted in increased activation of the hypoxia-induced factors (HIF-1/2) [7, 22]. The direct effect of overloading HIFs is the activation of targeted downstream genes (e.g., vascular endothelial growth factor (VEGF) and platelet-derived growth factor (PDGF)), which participated in various key biological processes including angiogenesis, glycolysis, lipogenesis, and many other metabolic pathways [30]. In another way, like many other cancers, the PI3K/Akt/mTOR pathway was also confirmed to be dysregulated and associated with aggressive tumor behaviors and poor prognosis in RCC. Resulted by excitation of PI3K and Akt, inactivation of tumor suppressor PTEN, mutation of VHL gene or stimulation from growth factors, activation of mTOR could finally lead to the aberrant accumulation of HIF-1α and HIF-2α [24]. Consequently, based on the solid understanding of VHL gene and key molecules’ function, RCC therapeutic management has greatly benefited from the derived targeted therapy. Various small molecular agents that target VEGFR/PDGFR (e.g., sunitinib, lenvatinib, pazopanib, axitinib and sorafenib), VEGF (e.g., bevacizumab), mTOR, and the MET and AXL tyrosine-protein kinase receptors [1]. Among them, mTOR has been confirmed to be indispensable and the representative targeting agents are temsirolimus, everolimus and ridaforolimus [24].

In the present study, we focused on a novel synthetic small molecular agent, NVP-BEZ235, that was designed to treat RCC by simultaneously inhibiting the activity of PI3K and mTORC1 in PI3K/Akt/mTOR pathway. In previous clinical trials on other solid tumors, NVP-BEZ235 was tested in several Phase I or I/II trials as a single agent or in combination with chemotherapy or other targeted agents [4, 14, 39]. In addition, it was also tested in combination with mTORC1 inhibitor everolimus in patients with solid tumors to verify its safety in vivo [23, 38]. Yet, there is still a lack of complete clinical trials performed on patients with RCC to provide dependable evidence for its clinical practicality. Beginning in 2011, sponsored by Memorial Sloan Kettering Cancer Center (MSKCC), the only (to our best knowledge) NVP-BEZ235 Phase I/II trial on patients with advanced RCC was conducted to investigate the maximally tolerated dose (MTD). During the trial, treatment with BEZ235 was poorly tolerated, with 50% of patients developed grade 3–4 adverse events, and it also limited the objective assessment of the drug’s effectiveness in RCC patients. Ultimately, the trial was forced to terminate given the number of observed toxicities and the difficulty with patient retention in the dose escalation portion [3, 36]. Nevertheless, from the perspective of efficacy, NVP-BEZ235 showed satisfactory laboratory antitumor effects in RCC cell lines through dual PI3K/mTOR inhibition. Roulin et al. found that no matter in vitro or in vivo, the combination of NVP-BEZ235 and sorafenib was more effective than each compound alone to repress tumor growth in ccRCC [28]. Meantime, Daniel et al. conducted in vitro and in vivo experiments to found that, compared with rapamycin, NVP-BEZ235 could induce growth arrest more effectively in RCC cell lines and it showed greater efficacy in suppressing tumor growth [6]. Combining the clinical and laboratory outcomes, we chose to explore the persistence of NVP-BEZ235 efficacy and the underlying mechanisms of drug resistance in ccRCC, aiming to determining its value in further research from another view.

To begin with, it was the construction of NVP-BEZ235-resistant ccRCC cell lines. It could be considered as the vital initiating step of the research because the judgement of resistance formation at different concentration is not objective enough and it could lead to distinct results. Clinically, after receiving the standard treatment of VEGF- and mTOR-targeted agents for a median time of 6–15 months, patients tend to show resistance of varying degrees and no longer benefit from the targeted therapy [21]. Previous studies provide different opinions on the involvement of this process. In the field of the most frequently investigated TKI, sunitinib, Sakai et al. established sunitinib-resistant RCC cell line (ACHN-R) which had an IC50 of about fivefold higher than the parental cells and detected the alterations of major signal transduction pathways associated with resistance [29]. Peng et al. considered the IC50 fold-change of more than 2 as the symbol of the sunitinib resistance in ccRCC cell lines [25], which was consistent with our point of view in previous study [37]. Meantime, the resistance could be told from the significant proliferation gap of cells instead of the accurate IC50, as long as the cells display different growth capability under certain concentration of agent [9, 34]. In our study, we still took fold-change of IC50 more than 2 as the hallmark of resistance formation in 786-O (fold-change = 2.96) and Caki-1 (fold-change = 5.19) cell lines. In the following research, multiple methods of investigating the cell proliferation in vitro or tumor growth in vivo testified the insensitivity to NVP-BEZ235 of 786-O-Res and Caki-1-Res cells.

Our work mainly introduced a novel regulatory relationship between LncRNA-CHKB-AS1 and the PI3K/Akt/mTOR pathway, which was mediated by MAP4, in the involvement of resistance to NVP-BEZ235. mTOR is a serine/threonine kinase with 2 distinct complexes: mTORC1 and mTORC2, and the former is confirmed to be sensitive to rapamycin. mTORC1 can specifically bind to the FK506 binding protein 12 (FKBP12)-rapamycin complexes and, once binding, its kinase activity could be inhibited [32]. mTORC1 can directly phosphorylate the major downstream targets including ribosomal subunit S6 kinase (p70S6K) and eukaryotic translation initiation factor 4E (eIF4E) binding protein (4E-BP1), through which signalings mTORC1 participate in the regulation of cell growth, protein translation and cellular metabolism [12]. In renal cell carcinoma, the PI3k/Akt/mTOR axis was confirmed to promote the aggressiveness of tumor and poor prognosis of patients via various mechanisms [24]. For the reason, agents that specifically target mTOR plays an imperative role in the landscape of small molecules targeted agents and showed meaningful efficacy. By applying lncRNA sequencing technique, we found that LncRNA-CHKB-AS1 was significantly dysregulated in NVP-BEZ235-resistant RCC cell lines. Then, solid cellular and animal experiments helped uncovering the promotive role of CHKB-AS1 in resistance formation via hyperactivating the phosphorylation of Akt and mTOR, which could be seen as the confrontation of the antitumor effects of NVP-BEZ235. Further molecular approaches revealed the significant RNA binding protein of CHKB-AS1, MAP4. Microtubule-associated protein-4 (MAP-4) belongs to MAP2/Tau family including vertebrate proteins MAP2, MAP4 and Tau, who share a conserved carboxy-terminal domain containing microtubule-binding repeats. MAP4 holds microtubule-stabilizing activity and has been proposed to participate in regulating mitotic microtubule dynamics during metaphase in cells [8]. Over years, MAP4 was narrowly reported to be associated with cancer progression or treating response. Yang et al. found that the sensitivity of paclitaxel can be enhanced by silencing several kinases to dysregulate the phosphorylation of MAP4 to increase the microtubule stability in ovarian cancer [41]. Meantime, the phosphorylation of MAP4 has been proved to play an indispensable role in numerous pathological processes, including endothelial cell and epidermal keratinocyte migration and proliferation [43, 44], pathological cardiac remodeling [19], cardiac microvascular density alteration [10], and mitochondrial dysfunction [18]. Among all studies focusing on MAP4 functioning, Narendra et al. investigated its key role in affecting the activity of PI3kα and the following PI3k-Akt signaling for the time [35]. It was found that MAP4 controlled the interaction of PI3Kα with activated receptors at endosomal compartments along microtubules, and loss of MAP4 results in the loss of PI3Kα as well as PI3K–Akt signaling downstream. The study creatively defined a novel mechanism for spatial control of PI3K–Akt signaling at internal membrane compartments associated with the microtubule network. The substantial discovery from Narendra et al. granted the finding in our work reasonable interpretations for molecular mechanism, which was, the spatial stability of PI3k was dependent on MAP4-mediated microtubules working network, and the downstream targeting of PI3k signaling including Akt/p-Akt-mTOR/p-mTOR and vital genes (e.g., cyclin B, cyclin D) could be significantly regulated by MAP4 accordingly.

In the clinical context, our findings on CHKB-AS1's role in NVP-BEZ235 resistance in renal carcinoma have significant implications. One potential application is in the optimization of NVP-BEZ235 therapy. By monitoring CHKB-AS1 levels, clinicians could identify patients more likely to benefit from this treatment or those who may require an alternative approach due to potential resistance. Moreover, our study opens avenues for developing combination therapies. For instance, combining NVP-BEZ235 with agents that target CHKB-AS1's regulatory pathways could enhance treatment efficacy. Such combinations might counteract the resistance mechanisms mediated by CHKB-AS1, thereby improving clinical outcomes for renal carcinoma patients. Additionally, given the role of the PI3K/Akt/mTOR pathway in renal carcinoma, our findings suggest that targeting this pathway, along with CHKB-AS1, could be a promising strategy. This approach may involve novel pharmacological inhibitors that specifically disrupt the CHKB-AS1-MAP4 interaction, potentially reducing the phosphorylation activity that leads to resistance.

In our study, we have highlighted the roles of CHKB-AS1 and MAP4 in modulating the PI3K/Akt/mTOR pathway and contributing to NVP-BEZ235 resistance in renal carcinoma. However, a more comprehensive understanding of their precise regulatory mechanisms remains essential. Future investigations should focus on unraveling the detailed molecular interactions and the specific regulatory roles these molecules play in resistance development. This deeper exploration could involve studying post-translational modifications of proteins involved in the pathway, the potential involvement of other co-factors, and the broader molecular network impacted by CHKB-AS1 and MAP4. Such mechanistic insights could unveil new therapeutic targets and strategies, enabling the development of more effective treatments for renal carcinoma. By understanding these complex molecular interactions, we can move closer to overcoming drug resistance and improving patient outcomes.

Overall, the present study found a vital signaling axis CHKB-AS1-MAP4-PI3K/Akt/mTOR and it enormously contributed to the formation of resistance to NVP-BEZ235 in ccRCC, badly impairing the antitumor efficacy of NVP-BEZ235. The inevitable insensitivity of small molecular targeted drugs in short- or long-term treatment has been a clinical dilemma in RCC patients for years. Attempts to discover the underlying mechanisms and improve the therapeutic effects of targeted agents are always urgently needed. We hope this finding could provide novel insights into understanding the resistance formation of NVP-BEZ235 and contribute to further clinical trials in the future.

## Conclusion

Our work found a new mechanism under NVP-BEZ235 resistance in ccRCC regulated by CHKB-AS1-MAP4-PI3k/Akt/mTOR axis. Due to the inevitable flaw, we concluded that this agent was not proper to apply in clinical treatment for ccRCC patients.

### Supplementary Information

**Additional file 1.** Differentially expressed lncRNAs.

**Additional file 2: Figure S1.** GSVA analysis and evaluation of transfection efficiency. A) GSVA analysis of patients with high and low CHKB-AS1 expression. B-I) Evaluation of transfection efficiency.

## Data Availability

The dataset used in this study can be found in the TCGA database: https://www.cancer.gov/about-nci/organization/ccg/research/structural-genomics/tcga.
