# Supplementary figures and images for: CHKB-AS1 enhances proliferation and resistance to NVP-BEZ235 of renal cancer cells via regulating the phosphorylation of MAP4 and PI3K/AKT/mTOR signaling
Source: Eur J Med Res. 2023 Dec 14;28:588. doi: 10.1186/s40001-023-01558-w (PMC10720114; doi:10.1186/s40001-023-01558-w)

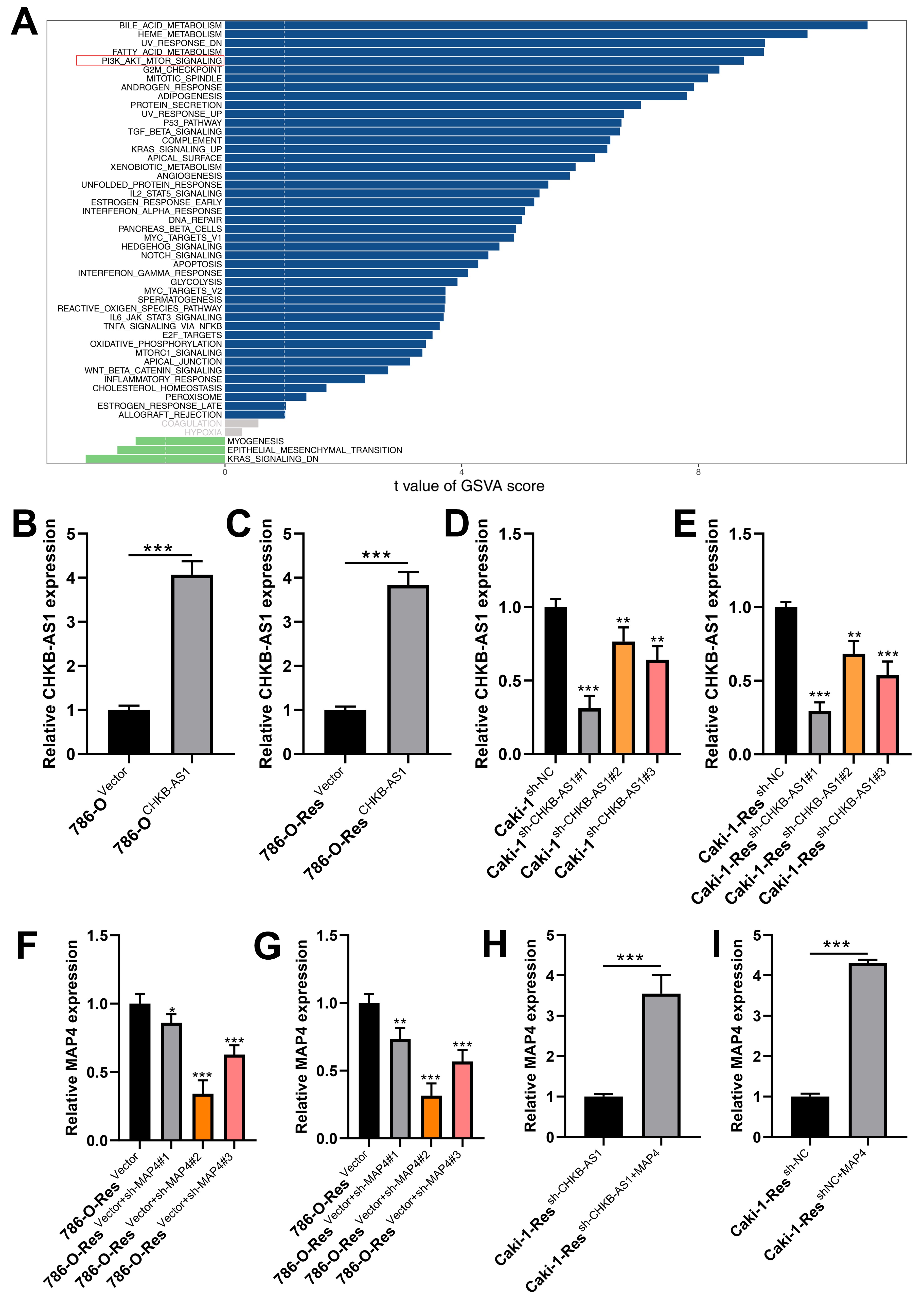

Supplement: Supplementary file 2 — Additional file 2: Figure S1. GSVA analysis and evaluation of transfection efficiency. A) GSVA analysis of patients with high and low CHKB-AS1 expression. B-I) Evaluation of transfection efficiency. [file 40001_2023_1558_MOESM2_ESM.tif]
